# Brazil’s Actions and Reactions in the Fight against COVID-19 from January to March 2020

**DOI:** 10.3390/ijerph18020555

**Published:** 2021-01-11

**Authors:** Ana Szylovec, Isis Umbelino-Walker, Brittany Nicole Cain, Hoi Tung Ng, Antoine Flahault, Liudmila Rozanova

**Affiliations:** 1 Institute of Global Health, University of Geneva, 1211 Geneva, Switzerland; brittany.cain@etu.unige.ch (B.N.C.); hoi-tung.ng@etu.unige.ch (H.T.N.); Antoine.Flahault@unige.ch (A.F.); Liudmila.Rozanova@unige.ch (L.R.); 2 Athena Institute, Faculty of Science, VU University Amsterdam, 1081 HV Amsterdam, The Netherlands; i.c.umbelinowalker@student.vu.nl

**Keywords:** COVID-19, Brazil, SARS-CoV-2, health policy, global health, public health

## Abstract

The outbreak of the novel coronavirus SARS-CoV-2 and the disease it causes, COVID-19, which emerged in 2019, was identified by the World Health Organization as a public health emergency of international concern. Brazil actively responded to contain the virus. This case study aims to examine Brazil’s response to COVID-19 by investigating the country’s actions and reflecting upon the outcomes throughout January and March 2020. The data collection strategy included gathering data from the country’s intergovernmental organization’s official website, epidemiological bulletins, and news reports, guided by intersectoral and interdisciplinary themes. Although the highest incidence rates were in the most rich and populated region in Brazil, it was the poorest region that had the highest case fatality rate. Nevertheless, Brazil took several non-pharmaceutical measures to control and mitigate the spread of the virus. However, the strategy seems to have failed to consider regional and social inequalities. The actions of the health minister were undermined by a conflicting discourse between the minister and the president. The outbreak of COVID-19 added an extra burden on the country’s healthcare system and the existing economic crises; exacerbated the inherent social, political, and economic challenges; and exposed the country’s contradictions.

## 1. Introduction

On 31 December 2019, the World Health Organization (WHO) was alerted to a cluster of pneumonia patients in Wuhan City, Hubei Province of China. On 7 January 2020, a novel coronavirus was identified as the cause of pneumonia. The virus was then named SARS-CoV-2 and the coronavirus disease was titled COVID-19 [1]. Epidemiological evidence shows that there was a human-to-human transmission of SARS-CoV-2. The virus was identified in environmental samples from a live animal market in Wuhan, and some human cases were epidemiologically linked to this market. However, the precise zoonotic origin is still uncertain [2]. On 30 January, the WHO declared the outbreak to be a public health emergency of international concern (PHEIC). With more than 118,000 cases in 114 countries, and 4291 deaths as of 11 March, the WHO announced that COVID-19 is characterized as a pandemic, the first caused by a coronavirus. The WHO called on all countries to activate and scale up their emergency response mechanisms, and remarked on the importance of balancing between protecting health, minimizing economic and social disruption, and respecting human rights [3].

Developing countries, which have limited fiscal and monetary capacity, face unique challenges not only in having the resources to respond to the pandemic but also in dealing with the consequences that go beyond health impacts. Brazil, in particular, as a continental country, presents great social, economic, and structural disparities within the country. In addition, before the pandemic, the country was already facing economic and political crises. Despite that, Brazil has a history of being an active actor in international cooperation for health [4]. The Brazilian leadership can be exemplified with the establishment of a strategic mass vaccination program, the effective HIV/AIDS program, leadership in the fight against tuberculosis, and more recently, the detection and containment of the congenital Zika virus syndrome emergency [5].

Following the announcements made by the WHO, Brazil actively prepared for the pandemic of COVID-19. On 22 January, the Ministry of Health (MoH) established an Emergency Operations Centre (COE) to coordinate actions and prepare the health system to respond to possible cases. The Centre was composed of three main institutions: Fiocruz (Oswald Cruz Foundation—Fundação Oswald Cruz), Anvisa (Brazilian Health Regulatory Agency—Agência Nacional de Vigilância Sanitária) and the Pan American Health Organization (PAHO). The COE led the technical capacity to respond to the pandemic in the country and Latin America by training health professionals and providing test and clinical practice guidelines [6].

As substantial progress and knowledge over the actions taken to control this unprecedented pandemic are needed, it is imperative to understand how developing countries respond to this global sanitary emergency. This case study aims to examine Brazil’s response to COVID 19 by investigating the country’s actions and reflecting upon the outcomes during January and March 2020.

## 2. Methodology

This is a single case study with an intrinsic design [7]. The unit of analysis is Brazil’s response to COVID 19 and is defined by the beginning of the country’s first measure to tackle the pandemic. Data collection involved unobtrusive measures—such as official (inter)governmental statements, legislation, and statistical information from official websites—and secondary data—such as epidemiological bulletins and news reports—that covered the study’s timeframe between January and March 2020. The first data points used and reproduced in this case study date to 22 January 2020, while the last data points date to 15 April 2020. Thus, the study was built in concomitance with the events, hence its peculiar “real-time” character. Much has happened in terms of data and outcomes, since the pandemic continues to unfold.

An intersectoral and interdisciplinary theme guided the data collection and the data analysis process. The guiding themes comprised the non-pharmaceutical measures taken, as well as the economic and social consequences of the pandemic in the country that are related to COVID 19. The analytical approach simply consisted of the description and analysis of the data. This strategy seemed appropriate since it enabled it to gain an understanding of the dynamics of this contemporary event, the pandemic in the Brazilian setting, within its real-life context.

## 3. Findings

### 3.1. Case Presentation

#### 3.1.1. Brazil’s Context

The Federative Republic of Brazil is the largest and most populous country in Latin America and is home to 210 million inhabitants. The country is composed of the partnerships of states, municipalities, and the federal district. They are all autonomous under the terms of the Constitution [8]. Although the country is unified, that does not mean that it is homogeneous. Brazil has several types of social inequalities, not only limited by factors, such as race or social position, but also regional differences.

The disparities between regions are historically rooted, and they shape the country, politically and economically [9]. Table 1 summarizes the indicators regarding the distribution of the population, GDP, and sanitation by region and state, and the human development index (HDI). The indicators display the inequalities between the regions. For instance, the southeast has a human development index (HDI) of 0.794 [10]. It is the most populated region, housing 42% of the Brazilian population, with 22% residing in the state of São Paulo only [11,12]. The area is responsible for more than 50% of the GDP and employs 45% of Brazil’s labor force [10,12]. In contrast, the north has an HDI of 0.730 [10], and it is the second less populated region in the country, with 8.77% Brazilians living there [12]. The differences in access to clean water between regions, states, and municipalities are also consistent with the country’s health conditions and are directly linked to the country’s social inequality. For instance, in the north, 57% of the population have access to clean water and only 10.5% have access to sewage collection service, while in the southeast, these indicators correspond to 91% and 79.2%, respectively [13].

Besides the socio-economic differences, the country also has a diverse climate. It extends from equatorial in the north and northeast, to subtropical in the centre-west and southeast, and temperate in the south.

Brazil also occupies the position of the 5th largest country, with a GDP of 1.91 trillion dollars. The services sector represented 76% of the Brazilian GDP in 2019, while the industrial and agricultural sectors represent 21% and 4%, respectively [14]. Despite an average economic growth of 4.5% during the 2006–2010 period and 2.8% during the 2011–2013 period, since 2014, Brazil has been facing economic crises. The origin of the economic crises was multifaceted and encompassed the decrease of commodities and issues of corruption and political uncertainties. This scenario limited the government’s ability to implement necessary fiscal reforms, leading to a decrease in consumption as well as investor confidence. During 2015–2016 the country faced a GDP contraction of 3.6% and 3.4%, respectively. However, since 2017, the country has shown signs of a slow recovery [9]. Although the unemployment rate in Brazil is around 11%, almost half of the working population (41%) is in informality and deprived of working rights [12].

#### 3.1.2. Brazil’s Health System

The Brazilian tax-based health system, known as the Unified Health System (SUS), was created in 1990, soon after establishing health as a right in the Constitution in 1988. The system was built based on the principles of universality, integrity, and equity, guaranteeing access to health for the entire population [15]. The system is decentralized, which grants independence for municipalities and states to carry out their healthcare policies with the support of the federal government. Thus, tripartite and bipartite intergovernmental commissions count on the participation of the federal government, states, and municipalities for decision making related to health policies [15,16]. The private sector may participate in the SUS on a complementary basis [15]. Therefore, the health services in Brazil have two faces, the public and private.

During the recession period, in addition to rising unemployment, inflation, poverty, and budget cuts that directly affected social programs, Brazil’s healthcare system reported delays in staff payment, lack of equipment and medicine, and increased demand for health services. In 2018, to restore fiscal sustainability, the government adopted Constitutional Amendment 95/2016, freezing public spending until 2026. This measure directly impacted the health of Brazil, since it made it impossible to increase investments in this sector [17].

**Figure 1:**
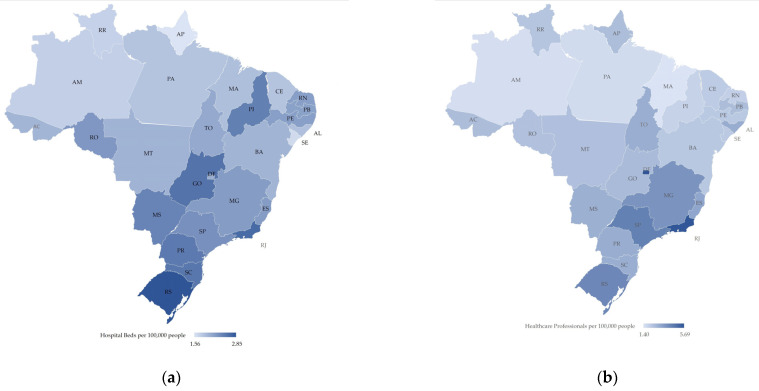
Map of Brazil, elaborated by the authors, with the distribution of healthcare resources by state: (**a**) Hospital Beds per 100,000 people ranging from 1.56 (light blue) to 2.85 (dark blue) (**b**) Healthcare Professionals (doctors and nurse) per 100,000 people ranging from 1.4 (light blue) to 5.69 (dark blue). Brazilian states acronym are: Acre (AC), Alagoas (AL), Amapá (AM), Amazonas (AP), Bahia (BA), Ceará (CE), DistritoFederal (DF), EspíritoSanto (ES), Goiás (GO), Maranhão (MA), MatoGrosso (MT), MatoGrossodoSul (MS), MinasGerais (MG), Pará (PA), Paraíba (PB), Paraná (PR), Pernambuco (PE), Piauí (PI), RiodeJaneiro (RJ), RioGrandedoNorte (RN), RioGrandedoSul (RS), Rondônia (RO), Roraima (RR), SantaCatarina (SC), SãoPaulo (SP), Sergipe (SE) and Tocantins (TO).

In general, public health services’ spatial distribution follows the historical trends of inequalities within the country [9]. Medium and high complexity equipment, such as Intensive Care Unit (ICU) beds and ventilators, remained concentrated mainly in capitals, metropolitan areas, and in a few regional centers. For instance, the southeast region has 53.4% of the total number of ICU beds in Brazil, while the north has 5.2% [18,19]. This scenario refers to equipment and human resources, where most of the intensive doctors, 57%, work in the southeast, while 3% work in the north [18]. Moreover, these disparities are further exacerbated when contrasting the amount of ICU beds available in the private sector. SUS holds 44% of the total ICU beds in the country, while the private’s sector has 56%, showing a disproportion, since only 24.6% of Brazilians own private insurance [20]. Figure 1 shows the number of hospital beds (a) and healthcare professionals (b) per 100,000 people in all Brazilian states, emphasizing the difference between states and regions [21].

#### 3.1.3. Epidemiological Situation

In March 2020, Brazil had had the first 30 days after the first case of COVID-19, which was confirmed on 26 February 2020. According to the Ministry of Health (MoH), until the end of March, the country confirmed 5717 cases and 201 deaths (Case Fatality ~3.5%; Incidence Rate ~2.7 per 100,000 people—the incidence coefficient per 100,000 inhabitants was calculated by the authors considering the IBGE (Instituto Brasileiro de Geografia e Estatistica—Brazilian Institute of Geography and Statistics) population projections for 2020 [8]). Most of the cases were concentrated in the southeast region (3406; 59.6%), followed by the northeast (875; 15.3%), south (672; 11.8%), centre-west (470; 8.2%), and north (294; 5.1%). More than half of the cases were concentrated in the states of São Paulo (40.9%) and Rio de Janeiro (708; 12.4%) [22]. The concentration of cases in the richest region was mainly due to (I) a high aerial network, which imported the first cases of COVID-19 from Italy; and (II) the population density, facilitating the dissemination of the virus.

The incidence rate followed a different trend, placing the southeast region at first with 3.9 per 100,000 population, followed by the centre-west (~2.9/100,000) and south (~2.2/100,000). Among the states, the Federal District had the highest incidence coefficient, with approximately 11 cases per 100,000 inhabitants. São Paulo had the second highest (~5.1/100,000), followed by Acre (~4.8/100,000), Ceará (~4.3/100,000), Amazonas (~4.2/100,000), and Rio de Janeiro (4.1/100,000) [8,18]. The two main factors that can explain this difference are the high mobility flow between national or international regions affected by the new coronavirus and containment measures adopted by the states.

**Figure 2:**
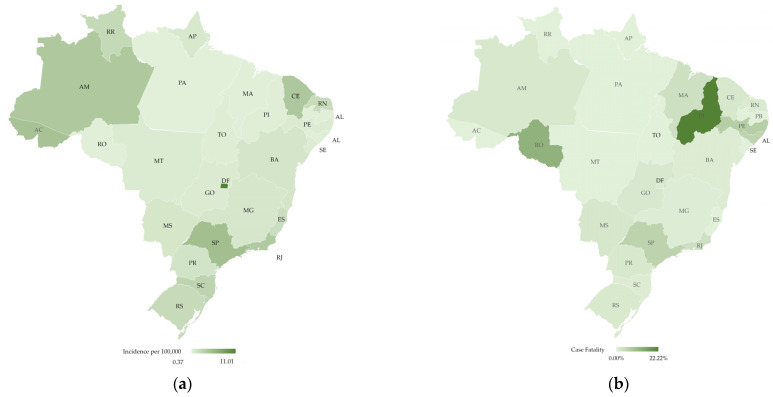
Map of Brazil with the distribution of COVID-19 cases and fatalities by state, elaborated by the authors: (**a**) Incidence of COVID-19 cases per 100,000 people ranging from 0.37 (light green) to 11.01 (dark green); (**b**) Case fatality (%) ranging from 0.00% (light green) to 22.22% (dark green). Brazilian states acronym are: Acre (AC), Alagoas (AL), Amapá (AM), Amazonas (AP), Bahia (BA), Ceará (CE), DistritoFederal (DF), EspíritoSanto (ES), Goiás (GO), Maranhão (MA), MatoGrosso (MT), MatoGrossodoSul (MS), MinasGerais (MG), Pará (PA), Paraíba (PB), Paraná (PR), Pernambuco (PE), Piauí (PI), RiodeJaneiro (RJ), RioGrandedoNorte (RN), RioGrandedoSul (RS), Rondônia (RO), Roraima (RR), SantaCatarina (SC), SãoPaulo (SP), Sergipe (SE) and Tocantins (TO).

The southeast region had the majority of deaths (161 deaths; case fatality ~4.7%), while the northeast had the second highest case fatality rate (2.5%) with 22 deaths. The south, centre-west, and north regions had nine, five, and four deaths, respectively [22]. Figure 2 shows the distribution of incidence per 100,000 people and case fatality rate per state.

The fatality rate per case highlights the fragility of the health system in the northeast and the pressure that the health system in the southeast was facing. For instance, Piauí (PI), even though it did not have a high incidence rate, presented a high case fatility rate. According to the MoH, more than 80% of the fatalities had at least one associated risk factor, with heart disease being the main one, followed by diabetes, pneumopathies, and neurological diseases. Moreover, approximately 85% of the cases were people over 60 years old [23].

The MoH established two risk groups: (I) people with health conditions and (II) health professionals. Individuals who were most at risk were over 60 years old, had severe or decompensated heart diseases, had pneumopathies, were immunosuppressed, had chronic kidney diseases in advanced stages (3, 4, 5), were diabetics, and were pregnant women. The government also identified health care professionals as a major concern because of the role they play in responding to the health emergency and because of the increasing numbers of confirmed cases among them due to lack of personal protective equipment (PPE). In a hospital in São Paulo, for instance, 348 employees were diagnosed with COVID-19, which corresponded to 2% of the employees in that hospital [23].

COVID-19 arrived in Brazil at the same period of its flu season, which began in mid-April until early September. Similar trends to influenza were expected by the MoH due to the agglomeration related to the winter season. In the south, which has a temperate climate, the peak usually happens in June and July. In the north, due to the rainy season, the biggest peak happens in March and April. Other regions presented an intermediary situation with less evident peaks during the winter [23].

Brazil’s laboratory capacity to perform tests for COVID-19 was considered insufficient by the ministry itself, leading to a number of cases being underreported. The network of Central Public Health Laboratories (Lacens), Fiocruz Institute and Evandro Chagas, was able to do 6700 tests a day [23]. Several models were developed to estimate the number of cases in the country. According to the mathematical model, based on the SIR model for disease spread and minimum t-norm, made by the Federal University of Pelotas (2020), the number of confirmed cases of COVID-19 in Brazil was 10,394 on 31 March 2020. In contrast, only 5717 were confirmed by the MoH at the end of March [24].

A different model, adapted from the Microscopic Markov Chain Approach (MMCA) metapopulation mobility model [21], considered the mobility and the demographical data for each municipality to capture the spread of COVID-19 [25]. As a result, the model, which did not include imported international cases, generated an indicator for each municipality, expressed by the risk of contracting the virus through local transmission [26]. The city of São Paulo, which held the index case, had a prediction of 0.04673% risk on 26 March, meaning that 46.73 people for every 100,000 individuals might be infected, either manifesting symptoms or not. The number of confirmed cases on the same date was 1052, which resulted in an incidence rate of 8.21 people for each 100,000 during the month of March [27]. On 26 March, the city reported 4621 suspected cases of COVID-19, waiting for testing confirmation [27]. Even though it seems the predictions overestimated the number of cases compared to the number of confirmed cases, they also showed the cities’ lack of ability to test for SARS-Cov-2.

Considering the fact that the ministry did not consider socio-economic characteristics in their reports and the high level of inequality between and within states in Brazil, there was a demand for mathematical models or analyses that include social vulnerability, mainly related to living conditions. Coelho et al. classified municipalities based on their vulnerability. The model considered urban indicators in education, health, and income distribution, highlighting the inequality between the north and south of the country. Their study showed that the regions in the north and north-east were more vulnerable to COVID-19 when compared to the other regions [19].

**Figure 3:**
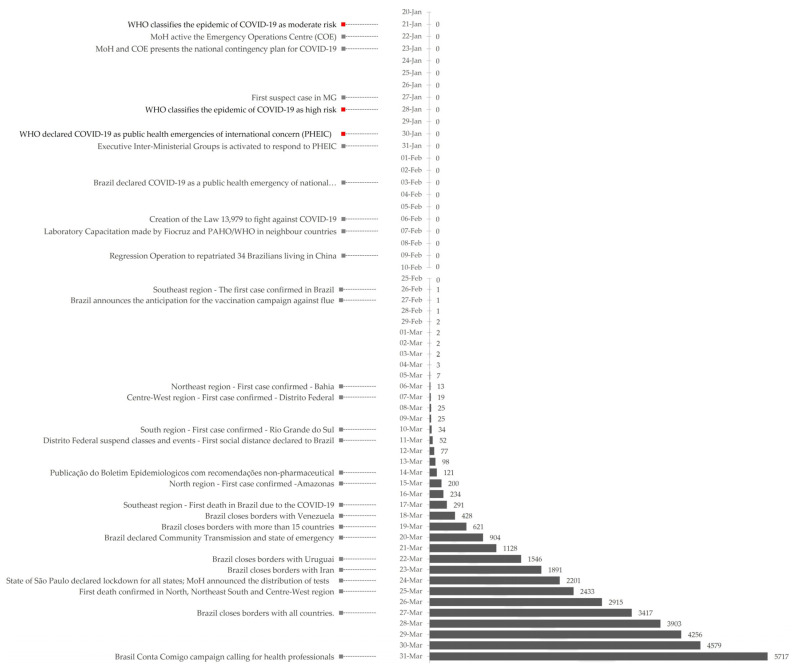
Timeline with measures taken by the Ministry of Health (grey) and the WHO (red) on the left with the evolution of accumulate cases of COVID-19 on the right.

Brazil faced great challenges during the first three months of this unprecedented pandemic given the country’s social-economic context, the epidemiological situation-early transmission phase, and the concomitant period of the flu season-and the health system conditions. This scenario highlighted the complexity of the response needed to the new coronavirus in Brazil. Figure 3 shows the evolution of cases in Brazil from 20 January to 31 March as well as the measures implemented by the Ministry of Health discussed in the “Non-Pharmaceutical Measures” session.

### 3.2. Management and Outcome

#### 3.2.1. Non-Pharmaceutical Measures

The Emergency Operations Centre (COE), after its creation, developed the “National Contingency Plan for Human Infection with the new COVID-19 Coronavirus,” which established three levels of response: alert, imminent danger, and public health emergency of national importance (PHENI). Each level of response defined the role of the institutions which make up the national healthcare system. On 3 February, Brazil declared the last level of response (PHENI) to allow the mission of repatriating 34 Brazil citizens living in Wuhan [6].

To assist the crisis, the Ministry of Health (MoH) announced the anticipation of influenza’s vaccination campaign. Vaccinations started on 23 March instead of the second half of April as was typically the case [28]. On 27 February, they declared that the campaign would be targeted to vaccinate the most vulnerable population, including children, elderly people, health professionals, and pregnant women. Their goal was to facilitate the differential diagnosis between COVID-19 and influenza and reduce the number of people seeking healthcare.

On 14 March, the MoH issued a publication providing recommendations on non-pharmaceutical intervention measures to be adopted by Brazilian cities and states to reduce the possibility of transmission of the virus [29]. In general, the MoH recommended promoting personal and public hygiene; the isolation of people with symptoms for 14 days; and the use of personal protective equipment for patients and health professionals. According to the publication, the MoH categorized COVID-19 cases into the local transmission and community transmission [29]. The former refers to the occurrence of a domestic case with an epidemiological link to a confirmed case, while the latter refers to a case without such a link. With this categorization, depending on the pandemic development and healthcare services capacity of a specific region, the MoH suggested respective non-pharmaceutical intervention measures for cities and states to contain the pandemic better.

Regarding areas with local transmission, apart from general personal and public hygiene, measures were focused on vulnerable groups, social contact restriction, patient referral procedure, and the reduction of unnecessary mass events. Regarding areas with community transmission, the MoH suggested social distancing measures for companies and education institutions (e.g., the use of virtual meetings, flexible working hours). It also provided recommendations for healthcare services, which included daily monitoring on COVID-19 cases, and quarantine measures to be adopted when reaching 80% of Intensive Care Unit (ICU) bed occupancy to ensure the necessary capacity needed for the pandemic response [29]. Despite the MoH’s general recommendation for municipalities and states, they may act differently in accordance with their capacity.

Starting from 17 March, several municipalities declared a state of emergency, such as Rio de Janeiro [30], followed by São Gonçalo and Guapimirim on 18 March [31]. On 19 March, the state Rio Grande do Sul prohibited interstate transport between itself and other Brazilian states to restrict travel [32]. On 20 March, Brazil’s Senate approved a presidential decree to declare the state of emergency at a national level. Under this measure, the government could waive fiscal targets and free up budget resources to combat the pandemic [33]. The MoH also declared on the same date the recognition of community transmission of COVID-19 throughout the national territory [34].

With 291 confirmed cases and the first death reported on 17 March, Brazil partially closed its border with Venezuela starting on 18 March for 15 days [35]. Health Minister Luiz Henrique Mandetta commented that Venezuela as a country was no longer able to provide healthcare. Hence, such measures were taken to alleviate the influx of Venezuelans overburdening Brazil’s health services. There was controversy over this measure, as the country did not close its border with other countries, which had more confirmed cases than Venezuela. Later on 19 March, Brazil also closed its land borders with Argentina, Bolivia, Colombia, French Guyana, Paraguay, Peru, and Suriname for 15 days to prevent the spread of the coronavirus [36]. On the same day, foreigners from several European and Asia Pacific countries were also restricted from entering the country by air for 30 days [37]. Starting from 22 March, a similar restriction also applied to Uruguay [38] and all foreigners entering Brazil over water [39] or air [40].

The MoH defended and advised social distance based on the recommendation of WHO to avoid the collapse of the healthcare system in Brazil. As such, all states were expected to adopt respective non-pharmaceutical measures aiming to help contain the pandemic; however, they were not legally biding to do so. In March, states such as Distrito Federal (11 March) and São Paulo (24 March) declared social distancing measures, but there were still some states that had not adopted them yet. The differences in the healthcare capacity between regions posed a challenge to the federal government. To cope with the pandemic, the Brazilian federal government installed measures to maintain enough healthcare inputs such as: personal tests, healthcare workforce, protective equipment (PPE), and hospital equipment (ICU beds and ventilators) [23].

On 24 March, it was reported that the Ministry of Health would expand the testing distribution to 22 million. Two types of tests were either purchased or donated. The first test was an RT-PCR test. This test identifies the virus while it is present in the body, and is typically used for patients hospitalized with severe symptoms. The second test consisted of serological assays. These respond to a reaction of the immune system to the virus, and they were typically used for healthcare workers at an increased risk of coming in contact with SARS-CoV-2 because of their close contact with patients. [41]. Brazil also established public–private partnerships aiming to guarantee the production of these tests [23].

To mobilize the health workforce to respond to the emergency situation for outpatients and hospital services, the MoH established the Strategic Action “O Brasil Conta Comigo” on 31 March, calling for health care professionals. The objective of the Strategic Action was registering and training health care professionals to combat the pandemic. Under this, all health professionals qualified to work in the national territory must register with the MoH through their respective professional councils. Training in the form of distance courses was provided to health professionals [42].

The MoH had been purchasing and redistributing PPE for the region to guarantee resources for the entire country. Most of the resources were allocated according to the region’s needs. Therefore, around 39% of all resources were in the southeast, followed by the northeast (28%), north (11%), and south (9%) [41]. On 17 March, the Chamber of Deputies approved the measure of prohibiting the export of health-related products necessary to contain the virus to prevent their shortage in the domestic market. Such products include PPE, such as latex gloves, goggles, and surgical masks, and hospital equipment, such as hospital beds and multi-parameter monitors [43].

In March, the MoH stated that Brazil’s health system would collapse by the end of April [44]. For instance, the shortage of hospital beds was worrying. Under the collaboration with Fiocruz, in March, the institution started to build a 200-bed hospital of intensive and semi-intensive care for COVID-19 patients [6]. States also built campaign hospitals, such as Rio de Janeiro [45] and São Paulo, which transformed a soccer stadium into a hospital [46].

Through all measures implemented by the Ministry of Health, the federal government aimed to reduce the transmission of COVID-19 by promoting technical information to the states, monitoring available resources, and researching existing questions. The government also recognized the gap between announcing resources’ availability and making them feasible for the states. Consequently, the recommendation for the states and municipalities was to implement the “extended social distancing until the health inputs and teams are available in sufficient quantity, in order to promote, with security, the transition to the strategy of selective social distancing” [23] (p.20). Until 31 March, no technical recommendations were provided by the MoH related to people living in poor living conditions, such as in favelas and those without access to water, and indigenous Brazilian communities.

#### 3.2.2. Economy

The scenario of uncertainty produced by the COVID-19 pandemic in the world economy was also evident in Brazil’s economy, which previously presented a gradual resumption of growth. These uncertainties are reflected in a dollar appreciation of +15.4% and a drop on the stock market of −35.8% and S&P500 of −24% on the financial market. In March, these impacts caused Brazil to break many records in its economy. Among them, the highest dollar price since the creation of the real coin and the highest number of circuit breakers (four) in one week on the stock exchange. The devaluation of Brazilian companies on the financial market also represented macroeconomic expectations, which followed the same trend [47].

The Institute of Applied Economic Research (IPEA) predicted a significant drop in economic activity during March. Brazil was expected to go through a recession in the first half of 2020. Although in the first quarter, the prediction was only a 0.2% reduction in the economy, compared to the previous three months, in the second quarter, they forecasted a fall of 2.13% in GDP [47]. In March, the central bank of Brazil (Bacen) predicted for the total economic growth of 2020, a fall from 2.2% to −0.48% [48]. This fall was mainly due to the increase in domestic costs, international shocks in the national economy, and a decreased in consumption.

The domestic costs related to the pandemic can be classified into direct and indirect costs. Among the indirect costs, there were the consequences of the COVID-19 containment measures, such as the decreased supply of services due to isolation measures. Among the direct costs were the loss of labor force—related to increased mortality—sick leave—due to COVID-19—and the government increasing spending on the health sector. International shocks refer to the reduction of capital flows and the world economy’s slowdown, which impacted exports and imports [47].

The fact that the region most affected by COVID-19 is also the region responsible for most of the country’s GDP and economic activity [49] put pressure on Brazil to adopt daily countercyclical macroeconomic measures [47]. Thus, despite the constitutional amendment 95, establishing a freeze on government spending, the ministry of economics announced extraordinary credits for different sectors to not be computed by the amendment. Besides the health sector, the sectors most affected by the COVID-19 pandemic were the services sector, except for services that are considered essential, and the informal labor sector, which contains 41% of the Brazilian labor force [47].

In order to respond to the prospects of increased unemployment and falling income, the government adopted a monetary policy of “reducing interest and compulsory rates and measures to expand credit with the temporary relaxation of prudential rules” [47] (p. 2). In March, different fiscal measures adopted by Brazil reached R$ 280.1 billion and aimed at reducing the impact on income, employment, production, and companies, as well as increasing social protection. Table 2 summarizes all the fiscal measures.

R$ 46 billion (~9.01 billion USD) was put aside for the elderly population. Workers were supported with R$ 32.8 billion (~6.4 billion USD) through the anticipation of social benefits, while 45 billion (~8.8 billion USD) was made available to workers in the informal sector. The government allowed workers to access their social benefits if they take sick leave over 15 days due to the coronavirus. Other efforts included putting aside R$77.7 billion (~15.21 billion USD) to benefit companies in general and investing R$19.9 billion (~3.9 billion USD) into the SUS. The government has also offered to assist states and cities with R$ 50.6 billion (~9.91 billion USD) by delaying the deadline for them to pay their debts to the federal government, passing on financial resources, and offering loan credits. In order to decrease the spending in the health sector, the government decided to reduce to zero the tax to imported hospital products until the end of the year and to exempt IPI (Industrialized Products Tax) temporarily for goods needed to fight COVID-19 [47,50].

#### 3.2.3. Social and Political Disruption/Media Coverage

Health Minister Luiz Henrique Mandetta tried to implement WHO’s recommendations and learn from the international experience. Although the Ministry of Health remained consistent in its approach to slowing the spread of COVID-19, the main critique over its actions lies over the impracticalities of adopting these measures in a country surrounded by inequalities [9]. The socially vulnerable population did not have the conditions to adequately self-isolate while living in overcrowded slums and having job positions that cannot be done from home. Moreover, the lack or absence of sanitation made it even harder to regularly follow the recommendation of washing hands. Furthermore, the fragile economic situation of this community mades it difficult to purchase basic hygiene supplies. Fiocruz and civil society led a movement to draw authorities and society’s attention on this problem [51]. However, until 31 March, social vulnerability was not included as an indicator on any of the epidemiological bulletins, and no epidemiological model predictions had been made of how the virus would spread in such circumstances, therefore hampering the country’s ability to take adequate and urgent measures [52].

In addition to the country’s complex social situation, there were also discrepancies between the Ministry of Health’s recommendations and measures and the presidency. Mandetta’s voice was overshadowed by President Jair Bolsonaro, who called COVID-19 a “fantasy” and accused the media of promoting hysteria over the population by its constant coverage of the subject. President Bolsonaro received backlash after mingling with supporters following his return from a trip with the United States’ President Donald Trump, in which more than 20 members of the trip tested positive for COVID-19 [53]. Although the president took a more severe angle by announcing that protestors should reconsider protesting the National Congress and Supreme Federal Court to support a more authoritarian government, he then sent them a WhatsApp message congratulating demonstrators for protesting in support of his ideology [54]. Bolsonaro, after having been tested three times for COVID-19, refused to make his results public until 31 March [55].

President Bolsonaro seemed to be more concerned about the economy than the serious health threat that COVID-19 presents. The president’s official pronouncements from 24 March called COVID-19 a “little cold.” [56]. His announcements contradicted several of the MoH’s measures and recommendations and escalated the tensions between Bolsonaro and Mandetta. For instance, Bolsonaro defended the use of chloroquine and extolled its suspected positive effects. In contrast, Mandetta emphasized that further studies were still needed to understand the medicine’s full effects. Another controversial position that the president took was the call for vertical isolation, where only the most vulnerable population should remain isolated. The MoH, on the contrary, reinforced the need to standardize isolation measures in the country when necessary. The minister’s opinion was supported by most of the scientific and medical community [57,58,59]. These frictions within the executive power put the minister’s position at risk, and the president threatened to fire him.

Bolsonaro also presented a clash with several governors and mayors who installed restrictive measures for the circulation of people in an attempt to reduce the spread of the disease [60]. The president advocated a relaxation of the social isolation measures implemented in the states. This dispute with governors led the president to promote propaganda called “Brazil cannot stop” to encourage the population to return to their regular social and economic activities. However, the Ministry of Justice prevented the government from promoting this propaganda, claiming that the campaign incites behaviors that are not based on technical guidelines [61]. Nevertheless, there were some states that chose to remain faithful to Bolsonaro’s position and refused to adopt measurements of social distancing [62].

This split between the powers was also reflected in the population. A poll conducted in São Paulo revealed that Brazilians’ overwhelming majority supported strict measures to slow the spread of COVID-19 [63]. The president’s refusal to take COVID-19 as a serious threat caused Brazilians to protest against his inaction by banging pans from their home’s window [53]. Although he was voted into office on the promise to boost Brazil’s economy, the people were much more concerned about the health threat COVID-19 posed. A petition was signed for more over than 1 million people asking the parliament to impeach the president [64]. Moreover, several political leaders sent an open letter to the president, asking him to resign [65]. Despite this, a part of the population was active on the streets in organized protests to support the president’s statements [66]. There was much controversy over the reluctance of certain groups to take restrictive care during this time. One church refused to close its doors, which was supported by President Bolsonaro [63]. Some 1500 prisoners escaped a semi-open prison in São Paulo after learning they will not be released for the holidays, and visits would be restricted [67].

The corporate side was also polarized. Although some companies helped either by donating money or supplies, most of the business side was against social distancing measures [68,69]. A Brazilian businessman stated that although Brazil would mourn those who die from COVID-19, Brazil should not shut down businesses, because he believed this would have a more significant impact on Brazilians than the spread of COVID-19 [70]. This argument proved to be fallacious. Even though the economic recession in Brazil, indeed, could contribute to deteriorating health conditions and increasing mortality, the investment in health and social protection addresses those issues, especially with regards to the most vulnerable populations, highlighting the need to strengthen health and social security [71].

Besides the internal conflicts, the COVID-19 pandemic also escalated some diplomatic tension between Brazil and China. The geopolitical dispute was initiated by a tweet from Eduardo Bolsonaro—son of the president and federal deputy—comparing the Chinese late response to communicate the virus emerging to the Chernobyl accident and, ultimately, blaming China for the pandemic. The tweet caused discomfort with the Chinese ambassador in Brazil, who demanded that the deputy and the Brazilian chancellor apologize to the Chinese government. After this event, several other members of the government started blaming China, calling COVID-19 the Chinese virus. However, other Brazilian politicians condemned their actions [72].

Brazil demonstrated internal and external tensions during January and March while trying to respond to the threat that COVID-19 posed to the pursuit of recovering the economic growth and, at the same time, the collapse of the health system. The debate and actions taken during this period were permeated by the historical and sociological inequalities inherent to the country and also by the polarized (geo)politics debates and positions on the issue.

## 4. Discussion

Once the WHO announced information related to COVID-19, Brazil reacted quickly to contain the pandemic in the country. Measures adopted since the establishment of the COE increased the healthcare system’s capacity to respond to the health emergency in collaboration between different sectors. At the same time, the arrival of the novel coronavirus in the country also exacerbated the inherent social, political, and economic challenges. For instance, although the highest incidence rates were in the most rich and populated region in Brazil, it was the poorest region that had the highest fatality rates. Furthermore, it exposed the contradictions of this continental country.

In response to the pandemic, the MoH recommended various non-pharmaceutical measures. They included a range of actions, from promotion and prevention to pandemic surveillance and coordination of healthcare resources and personnel. Those measures were aimed to avoid the collapse of a health system that has been underfinanced since 2014. On top of this, there were issues of different health capacities between regions as well as differences in health access between those with and without private health insurance.

One of the central contradictions of the measures proposed by the MoH was the classification of the risk groups. While the minister defined just the health professional and people with health conditions as at risk, the people living in poor conditions and the traditional communities remained without any guidelines. Social status, from January to March, was not considered in any of the epidemiological bulletins. The social distancing measures may have had the worst effect on the workers that needed the coming and going to guarantee their earnings. The restrictions may have harmed those living in the slums. With schools closing, these communities may have became even more overcrowded, putting at risk the population in these areas.

The biggest challenge that the country was facing was economical due to economic crises inherited since 2014. Nevertheless, the Ministry of Economy adopted several fiscal measures that englobe mainly helping companies and workers and assisting the poorest and the informal sector. Moroever, the federal government also repassed financial resources to the health sector to respond to the COVID-19 pandemic. Tackling social protection and the health sector can help increase health coverage and reduce the pandemic’s impact. However, given the uncertain scenery, it was unknown whether these measures would be enough and how long Brazil could sustain them.

Economic and political disruptions further exacerbated this alarming scenario. The absence of a coherent discourse between the president and the Ministry of Health, governors, and mayors caused political insecurity and confusion. The minister tried to steer the country towards the technical advice but was undermined by his president. These frictions and conflicting information resulted in polarization in all clusters in Brazilian society, from the top-level government to the ordinary citizen. This duality of visions hampered the containment of the virus in the country, since it brought forth difficulties in the compliance with health policies measures recommended in fighting the virus.

The main limitations of this research lay in its design. Although the real-time case study approach allowed to grasp a unique perception of the unfolding of the pandemic in Brazil, it also constrained and limited data collection. Moreover, external validity is low, and the study does not draw generalizations. It would be beneficial to investigate this period of the pandemic in Brazil from a different theoretical perspective, for instance, health equity perspective in public health governance, to contrast with the thematic analytical approach of this study.

## 5. Conclusions

This case study reported, in real-time, the dynamics of the COVID-19 pandemic in Brazil by exposing the outcomes from an intersectoral and interdisciplinary point of view. It seems that the COVID-19 pandemic posed unique challenges for developing countries. From January to March, Brazil’s experience showed that this pandemic crisis exacerbated political, social, and economic challenges that the country was already facing. However, Brazil also reaffirmed its leadership and coordination capacity, especially in fiscal and economic measures. This case study pointed to the need to include vulnerable populations and traditional communities while drawing emergency measures. Moreover, this case study exposed the importance of unified leadership when responding to a health crisis, including civil societies, the public sector, the private sector, government, and international organizations. More research is needed to continue the evaluation of Brazil’s response as well as the effect of the measures that were implemented (or not).

## Figures and Tables

**Table 1:** Human development index (HDI), GDP, population, and sanitation indicators by region and state.

State		HDI	GDP		Population			Sanitation	
		HDI	GDP ($100,000)	%	Population	%	Density (pop/km^2^)	Access to Clean Water (% pop)	Access to Sewage CollectionService (% pop)
**North**		**0.730**	**1,061,739.73**	**5.5%**	**18,430,980**	**8.8%**	**4.79**	**57.0%**	**10.5%**
RO	Rondônia	0.725	123,052.05	0.6%	1,777,225	0.8%	7.47	49.4%	4.9%
AC	Acre	0.719	42,002.74	0.2%	881,935	0.4%	5.37	47.1%	10.1%
AM	Amazonas	0.733	274,271.23	1.4%	4,144,597	2.0%	2.66	81.1%	10.0%
RR	Roraima	0.752	36,630.14	0.2%	605,761	0.3%	2.70	81.5%	51.7%
PA	Pará	0.698	442,054.79	2.3%	8,602,865	4.1%	6.91	45.6%	5.2%
AP	Amapá	0.740	46,013.70	0.2%	845,731	0.4%	5.94	34.9%	7.1%
TO	Tocantins	0.743	97,715.07	0.5%	1,572,866	0.7%	5.66	79.3%	26.4%
**Northeast**		**0.711**	**2,752,953.42**	**14.3%**	**57,071,654**	**27.2%**	**36.77**	**74.2%**	**28.0%**
MA	Maranhão	0.687	268,983.56	1.4%	7,075,181	3.4%	21.46	56.4%	13.8%
PI	Piauí	0.697	138,021.92	0.7%	3,273,227	1.6%	13.01	75.9%	14.4%
CE	Ceará	0.735	427,134.25	2.2%	9,132,078	4.3%	61.33	59.0%	25.5%
RN	Rio Grande do Norte	0.731	183,479.45	1.0%	3,506,853	1.7%	66.41	87.1%	23.8%
PB	Paraíba	0.722	176,367.12	0.9%	4,018,127	1.9%	71.16	74.3%	36.1%
PE	Pernambuco	0.727	510,553.42	2.7%	9,557,071	4.5%	97.45	80.5%	27.5%
AL	Alagoas	0.683	149,076.71	0.8%	3,337,357	1.6%	119.86	74.6%	21.3%
SE	Sergipe	0.702	115,117.81	0.6%	2,298,696	1.1%	104.83	86.9%	25.5%
BA	Bahia	0.714	784,219.18	4.1%	14,873,064	7.1%	26.34	81.6%	39.5%
**Centre-West**		**0.790**	**1,903,865.75**	**9.9%**	**16,297,074**	**7.8%**	**10.15**	**89.0%**	**52.9%**
MS	Mato Grosso do Sul	0.766	293,065.75	1.5%	3,484,466	1.7%	9.76	86.4%	49.5%
MT	Mato Grosso	0.774	376,556.16	2.0%	2,778,986	1.3%	3.08	89.3%	35.6%
GO	Goiás	0.769	536,115.07	2.8%	7,018,354	3.3%	20.63	85.5%	46.4%
DF	Distrito Federal	0.850	698,128.77	3.6%	3,015,268	1.4%	523.41	99.0%	89.3%
**Southeast**		**0.795**	**10,195,389.04**	**53.1%**	**88,371,433**	**42.1%**	**95.58**	**91.0%**	**79.2%**
MG	Minas Gerais	0.787	1,684,591.78	8.8%	21,168,791	10.1%	36.09	82.1%	72.1%
ES	Espírito Santo	0.772	375,397.26	2.0%	4,018,650	1.9%	87.22	81.2%	54.9%
RJ	Rio de Janeiro	0.796	2,079,065.75	10.8%	17,264,943	8.2%	394.62	90.5%	65.3%
SP	São Paulo	0.826	6,056,334.25	31.6%	45,919,049	21.9%	184.99	96.2%	89.8%
**South**		**0.796**	**3,275,479.45**	**17.1%**	**29,975,984**	**14.3%**	**51.97**	**90.2%**	**45.2%**
PR	Paraná	0.792	1,205,558.90	6.3%	11,433,957	5.4%	57.37	94.4%	71.4%
SC	Santa Catarina	0.808	817,060.27	4.3%	7,164,788	3.4%	74.84	89.1%	23.7%
RS	Rio Grande do Sul	0.787	1,252,860.27	6.5%	11,377,239	5.4%	40.39	86.4%	32.1%
**Brazil**		**0.778**	**19,189,427.40**	**100%**	**210,147,125**	**100.0%**	**24.69**	**83.6%**	**53.1%**

HDI: Human Development Index; GDP: Gross Domestic Product. Data in bold refers to the region’s of Brazil.

**Table 2:** Summary of the fiscal measures adopted by the government due to COVID-19.

Target Population	Value (R$ Billion)	Value (US$ Billion)
Elderly people	46.0	9.0
Workers in the formal sector	32.8	6.4
Workers in the informal sector	45.0	8.8
Companies	77.7	15.2
Health system	19.9	3.9
Cities	50.6	9.9

## Data Availability

Data sharing not applicable. No new data were created or analyzed in this study. Data sharing is not applicable to this article.

## References

[1] World Health Organization Naming the Coronavirus Disease (COVID-19) and the Virus that Causes It. https://www.who.int/emergencies/diseases/novel-coronavirus-2019/technical-guidance/naming-the-coronavirus-disease-(covid-2019)-and-the-virus-that-causes-it.

[2] World Health Organization 2019 Novel Coronavirus (2019-nCoV): Strategic Preparedness and Response Plan 2020. https://www.who.int/publications/i/item/strategic-preparedness-and-response-plan-for-the-new-coronavirus.

[3] World Health Organization WHO Director-General’s Opening Remarks at the Media Briefing on COVID-19–11 March 2020. https://www.who.int/dg/speeches/detail/who-director-general-s-opening-remarks-at-the-media-briefing-on-covid-19---11-march-2020.

[4] Buss P. (2011). Brazil: Structuring cooperation for health. Lancet.

[5] Ventura D.d.F.L., Ribeiro H., Giulio G.M.d., Jaime P.C., Nunes J., Bógus C.M., Antunes J.L.F., Waldman E.A. (2020). Challenges of the COVID-19 pandemic: For a Brazilian research agenda in global health and sustainability. Cadernos Saude Publ..

[6] Fiocruz The Fiocruz Response to the COVID-19. Fiocruz. https://fiocruz.tghn.org/coronavirus/.

[7] Yin R.K. (2017). Case Study Research and Applications: Design and Methods.

[8] Brasil Consitituição da Republica Federativa do Brasil 1988. http://www.planalto.gov.br/ccivil_03/constituicao/constituicao.htm.

[9] Albuquerque M.V.d., Viana A.L.d., Lima L.D.d., Ferreira M.P., Fusaro E.R., Iozzi F.L. (2017). Desigualdades regionais na saúde: Mudanças observadas no Brasil de 2000 a 2016. Ciência Saúde Coletiva.

[10] IPEA, PNUD (2019). Fundação João Pinheiro Radar IDHM: Evolução do IDHM e de Seus Índices Componentes no Período de 2012 a 2017.

[11] Instituto Brasileiro de Geografia e Estatística (IBGE) Projeções da População. https://www.ibge.gov.br/estatisticas/sociais/populacao/9109-projecao-da-populacao.html?=&t=o-que-e.

[12] Instituto Brasileiro de Geografia e Estatística (IBGE) Pesquisa Nacional por Amostra de Domicílios Contínua—PNAD Contínua. https://www.ibge.gov.br/estatisticas/sociais/trabalho/9173-pesquisa-nacional-por-amostra-de-domicilios-continua-trimestral.html?t=resultados.

[13] Panel de Saneamento Brasil. https://www.painelsaneamento.org.br/localidade?id=0.

[14] Instituto Brasileiro de Geografia e Estatística (IBGE) Balanço de Contas Nacionais. https://sidra.ibge.gov.br/tabela/1846#/n1/all/v/all/p/-1/c11255/90687,90691,90696,90705,90706,90707,93404,93405,93406,93407,93408,102880/l/v,,c11255+t+p/resultado.

[15] Brasil, Ministério da Saúde Sistema Único de Saúde (SUS): Estrutura, Princiípios e Como Funciona. https://www.saude.gov.br/sistema-unico-de-saude.

[16] Castro M.C., Massuda A., Almeida G., Menezes-Filho N.A., Andrade M.V., de Souza Noronha K.V.M., Rocha R., Macinko J., Hone T., Tasca R. (2019). Brazil’s unified health system: The first 30 years and prospects for the future. Lancet.

[17] Watts J. (2016). Brazil’s health system woes worsen in economic crisis. Lancet.

[18] Associação de Medicina Intensiva Brasileira (AMIB) (2016). Censo AMIB 2016.

[19] Coelho F.C., Lana R.M., Cruz O.G., Codeco C.T., Villela D., Bastos L.S., Piontti A.P.Y., Davis J.T., Vespignani A., Gomes M.F.C. (2020). Assessing the potential impact of COVID-19 in Brazil: Mobility, morbidity and the burden on the health care system. medRxiv.

[20] Agência Nacional de Saúde Complementar (ANS) Dados Gerais—ANS—Agência Nacional de Saúde Suplementar. https://www.ans.gov.br/perfil-do-setor/dados-gerais.

[21] Brazil, Ministério da Saúde TabNet Win32 3.0: E.1 Número de Profissionais de Saúde por Habitante. http://tabnet.datasus.gov.br/cgi/tabcgi.exe?idb2012/e01.def.

[22] Brazil, Ministério da Saúde, Painel Coronavírus. https://covid.saude.gov.br/.

[23] Centro de Operações de Emergência em Saúde Pública (COE) Boletim Epidemiológico 06–Doença pelo Coronavírus 2019. https://portalarquivos.saude.gov.br/images/pdf/2020/April/03/BE6-Boletim-Especial-do-COE.pdf.

[24] Universidade Federal de Pelotas (UFPel Gráfico da Evolução Temporal do Coronavírus. http://ccs2.ufpel.edu.br/wp/2020/03/26/grafico-da-evolucao-temporal-do-coronavirus-atualizacao-de-25-03-2020/.

[25] Cota W., Ferreira S.C. Risk map of COVID-19—Brazil. https://covid-19-risk.github.io/map/brazil/en/.

[26] Arenas A., Cota W., Gomez-Gardenes J., Gómez S., Granell C., Matamalas J.T., Soriano-Panos D., Steinegger B. (2020). A Mathematical Model for the Spatiotemporal Epidemic Spreading of COVID19. medRxiv.

[27] São Paulo Vigilância em Saúde. Boletins Epidemiológicos. https://www.prefeitura.sp.gov.br/cidade/secretarias/saude/vigilancia_em_saude/index.php?p=295572.

[28] Aquino V. Campanha de Vacinação Contra a Gripe Será Antecipada. https://www.saude.gov.br/noticias/agencia-saude/46449-campanha-de-vacinacao-contra-a-gripe-sera-antecipada.

[29] Centro de Operações de Emergência em Saúde Pública (COE) Boletim Epidemiológico 05–Doença pelo Coronavírus 2019. https://portalarquivos.saude.gov.br/images/pdf/2020/marco/24/03--ERRATA---Boletim-Epidemiologico-05.pdf.

[30] Rio’s State of Emergency Closes Christ Statue. https://www.smh.com.au/world/south-america/rio-s-state-of-emergency-closes-christ-statue-20200318-p54bge.html.

[31] G1 Rio Rio e Outros 5 Municípios do Estado Declaram Situação de Emergência Para Conter o Coronavírus. https://g1.globo.com/rj/rio-de-janeiro/noticia/2020/03/18/prefeitura-do-rio-declara-situacao-de-emergencia.ghtml.

[32] G1 RS Governo do RS Decreta Situação de Calamidade Pública Devido ao Coronavírus. https://g1.globo.com/rs/rio-grande-do-sul/noticia/2020/03/19/governo-decreta-situacao-de-calamidade-publica-no-rs-devido-ao-coronavirus.ghtml.

[33] Brasil, Regimento Interno do Senado Federal Decreto Legislativo n° 6, de 2020. http://www.planalto.gov.br/ccivil_03/portaria/DLG6-2020.htm#:~:text=DECRETO%20LEGISLATIVO%20N%C2%BA%206%2C%20DE,Art..

[34] Brasil, Ministério da Saúde/Gabinete do Ministro Portaria n° 454, de 20 de Março de 2020. https://www.in.gov.br/en/web/dou/-/portaria-n-454-de-20-de-marco-de-2020-249091587.

[35] Brasil, Presidência da República/Casa Civil Portaria n° 120, de 17 de Março de 2020. https://www.in.gov.br/en/web/dou/-/portaria-n-120-de-17-de-marco-de-2020-248564454.

[36] Brasil, Presidência da República/Casa Civil Portaria n° 125, de 19 de Março de 2020. https://www.in.gov.br/en/web/dou/-/portaria-n-125-de-19-de-marco-de-2020-248881224.

[37] Brasil, Casa Civil Portaria n° 126, de 19 de Março de 2020. https://www.in.gov.br/en/web/dou/-/portaria-n-126-de-19-de-marco-de-2020-248881688.

[38] Brasil, Casa Civil Portaria n° 132, de 22 de Março de 2020. https://www.in.gov.br/en/web/dou/-/portaria-n-132-de-22-de-marco-de-2020-249098650.

[39] Brasil, Presidência da República/Casa Civil Portaria n° 47, de 26 de Março de 2020. https://www.in.gov.br/web/dou/-/portaria-n-47-de-26-de-marco-de-2020-249861855.

[40] Brasil, Presidência da República/Casa Civil Portaria n° 152, de 27 de Março de 2020. https://www.in.gov.br/en/web/dou/-/portaria-n-152-de-27-de-marco-de-2020-250060288.

[41] Brasil, Ministério da Saúde Gráfico de Insumos. https://covid-insumos.saude.gov.br/paineis/insumos/painel.php.

[42] Brasil, Ministério da Saúde Portaria n° 639, de 31 de Março de 2020. https://www.in.gov.br/en/web/dou/-/portaria-n-639-de-31-de-marco-de-2020-250847738.

[43] Triboli P. Câmara Aprova Três Projetos Com Medidas de Combate ao Coronavírus. https://www.camara.leg.br/noticias/646163-camara-aprova-tres-projetos-com-medidas-de-combate-ao-coronavirus/.

[44] Al Jazeera Brazil’s Health System Will Collapse by April. https://www.aljazeera.com/news/2020/03/brazil-health-system-collapse-april-health-minister-200320190048602.html.

[45] Lisboa V. Hospital de Campanha Está Quase Pronto no Rio. https://agenciabrasil.ebc.com.br/saude/noticia/2020-04/hospital-de-campanha-esta-quase-pronto-no-rio.

[46] Bond L. Covid-19: São Paulo Ganhará Hospital de Campanha Dia 27. https://agenciabrasil.ebc.com.br/saude/noticia/2020-03/covid-19-sao-paulo-ganhara-hospital-de-campanha-dia-27.

[47] Instituto de Pesquisa Econômica Aplicada (IPEA) Carta de Conjuntura 46–1° Trimestre de 2020. https://www.ipea.gov.br/portal/index.php?option=com_content&view=article&id=35348.

[48] Brasil, Banco Central Focus—Relatório de Mercado 2019. https://www.bcb.gov.br/publicacoes/focus/27032020.

[49] Instituto Brasileiro de Geografia e Estatística (IBGE) Produto Interno Bruto—PIB. https://www.ibge.gov.br/explica/pib.php.

[50] Brasil, Ministério da Economia Medidas Econômicas Voltadas para a Redução dos Impactos do Covid-19. https://www.gov.br/economia/pt-br/index.

[51] Flaeschen H. Coronavírus Nas Favelas: “É Difícil Falar Sobre Perigo Quando há Naturalização do Risco de Vida”—ABRASCO. https://www.abrasco.org.br/site/outras-noticias/saude-da-populacao/coronavirus-nas-favelas-e-dificil-falar-sobre-perigo-quando-ha-naturalizacao-do-risco-de-vida/46098/.

[52] Carvalho M.S., Lima L.D.d., Coeli C.M. (2020). Ciência em Tempos de Pandemia.

[53] Phillips T. Brazilians Protest Over Bolsonaro’s Muddled Coronavirus Response. http://www.theguardian.com/world/2020/mar/22/brazilians-protest-bolsonaro-coronavirus-panelaco.

[54] Bevins V. (2020). In Brazil, Bolsonaro Gambles on a Coronavirus Culture War. N. Y. Rev. Books.

[55] Uribe G., Coletta R.D. Bolsonaro se Nega a Mostrar Exames Que, Segundo Ele, Deram Negativo Para Coronavirus. https://www1.folha.uol.com.br/poder/2020/03/bolsonaro-se-nega-a-mostrar-exames-que-segundo-ele-deram-negativo-para-coronavirus.shtml.

[56] Uol SP “Gripezinha”: Leia a Íntegra do Pronunciamento de Bolsonaro Sobre Covid-19. https://noticias.uol.com.br/politica/ultimas-noticias/2020/03/24/leia-o-pronunciamento-do-presidente-jair-bolsonaro-na-integra.htm.

[57] Monteiro W.M., Brito-Sousa J.D., Baía-da-Silva D., Melo G.C.d., Siqueira A.M., Val F., Daniel-Ribeiro C.T., Guimarães Lacerda M.V., Monteiro W.M., Brito-Sousa J.D. (2020). Driving forces for COVID-19 clinical trials using chloroquine: The need to choose the right research questions and outcomes. Rev. Soci. Bras. Med. Trop..

[58] Cortegiani A., Ingoglia G., Ippolito M., Giarratano A., Einav S. (2020). A systematic review on the efficacy and safety of chloroquine for the treatment of COVID-19. J. Crit. Care.

[59] Colbourn T. (2020). COVID-19: Extending or relaxing distancing control measures. Lancet Publ. Health.

[60] Cerioni C. Como os Estados Brasileiros Estão Agindo Para Conter o Coronavírus. https://exame.abril.com.br/brasil/como-os-estados-brasileiros-estao-agindo-para-conter-o-coronavirus/.

[61] Barroso M.R. Medida Cautelar na Arguição de Descumprimento de Preceito Fundamental 669 Distrito Ferederal. http://www.stf.jus.br/arquivo/cms/noticiaNoticiaStf/anexo/ADPF669cautelar.pdf.

[62] G1 SP Ao Menos 25 dos 27 Governadores Manterão Restrições Contra Coronavírus Mesmo Após Bolsonaro Pedir Fim de Isolamento. https://g1.globo.com/politica/noticia/2020/03/25/governadoras-reagem-ao-pronunciamento-de-bolsonaro-sobre-coronavirus.ghtml.

[63] Gielow I. Majority of Brazilians Are Afraid of Coronavirus and Support Containment Measures, Says Datafolha. https://www1.folha.uol.com.br/internacional/en/brazil/2020/03/majority-of-brazilians-are-afraid-of-coronavirus-and-support-containment-measures-says-datafolha.shtml.

[64] Change.org “Bolsonaro acabou”, Acreditam Assinantes de Petição Pelo Impeachment—CartaCapital. https://www.cartacapital.com.br/blogs/change-org/bolsonaro-acabou-acreditam-assinantes-de-peticao-pelo-impeachment/.

[65] Agostine C. Líderes da Oposição Pedem Renúncia de Bolsonaro. https://valor.globo.com/politica/noticia/2020/03/30/lideres-da-oposicao-pedem-renuncia-de-bolsonaro.ghtml.

[66] G1 SP Grupo Faz Carreata Contra Quarentena Imposta Por Doria em SP Devido ao Coronavírus. https://g1.globo.com/sp/sao-paulo/noticia/2020/03/27/grupo-faz-carreata-contra-quarentena-imposta-por-doria-em-sao-paulo.ghtml.

[67] Phillips T. “Come back Monday, OK?” Hundreds of prisoners escape in Brazil amid Covid-19 anger. http://www.theguardian.com/world/2020/mar/17/come-back-monday-ok-hundreds-of-prisoners-escape-in-brazil-amid-covid-19-anger.

[68] Uol SP Coronavírus: Madero, Havan, Giraffas: Empresários Criticam Medidas de Combate à Pandemia. https://economia.uol.com.br/noticias/redacao/2020/03/24/empresarios-coronavirus-o-que-dizem-criticas.htm?cmpid=copiaecola.

[69] Santana P. (2020). Empresas anunciam doações para combate a pandemia de coronavírus no Brasil; conheça as iniciativas. InfoMoney.

[70] Martins R.M., Demori L. (2020). Em áudio no WhatsApp, Bolsonaro dá parabéns a manifestações que devem aumentar propagação do coronavírus. Intercept.

[71] Hone T., Mirelman A.J., Rasella D., Paes-Sousa R., Barreto M.L., Rocha R., Millett C. (2019). Effect of economic recession and impact of health and social protection expenditures on adult mortality: A longitudinal analysis of 5565 Brazilian municipalities. Lancet Glob. Health.

[72] Fellet J. (2020). “Vírus chinês”: Como Brasil se inseriu em disputa geopolítica entre EUA e China sobre pandemia. BBC News Bras.

